# Chemical Composition and Bioactivity of Essential Oils from *Magnolia pugana*, an Endemic Mexican Magnoliaceae Species

**DOI:** 10.3390/molecules30183778

**Published:** 2025-09-17

**Authors:** Edison Osorio, José A. Vázquez-García, Paco Noriega, Ramón Reynoso-Orozco, Rosario Huizar, Mario Noa, Elisa Cabrera-Diaz, Lucía Barrientos-Ramírez, Hugo Cerda, Mario A. Ruíz-López

**Affiliations:** 1 Group of Research and Development in Science Applied to Biological Resources, Universidad Politécnica Salesiana, Av. 12 de Octubre N2422 & Wilson St., Quito 170109, Ecuador; pnoriega@ups.edu.ec; 2 Department of Botany and Zoology, Centro Universitario de Ciencias Biológicas y Agrícolas (CUCBA), Universidad de Guadalajara, Road Ing. Ramón Padilla Sánchez 2100, Las Agujas, Zapopan 45200, Jalisco, Mexico; jantonio.vazquez@academicos.udg.mx; 3 Department of Cell Biology, Centro Universitario de Ciencias Biológicas y Agrícolas (CUCBA), Universidad de Guadalajara, Road Ing. Ramón Padilla Sánchez 2100, Las Agujas, Zapopan 45200, Jalisco, Mexico; ramon.reynoso@academicos.udg.mx (R.R.-O.); mariadelrosario.huizar@academicos.udg.mx (R.H.); 4 Department of Public Health, Centro Universitario de Ciencias Biológicas y Agrícolas (CUCBA), Universidad de Guadalajara, Road Ing. Ramón Padilla Sánchez 2100, Las Agujas, Zapopan 45200, Jalisco, Mexico; mario.noa@academicos.udg.mx (M.N.); elisa.cabrera@academicos.udg.mx (E.C.-D.); 5 Department of Wood, Cellulose and Paper, Centro Universitario de Ciencias Exactas e Ingenierías (CUCEI), Universidad de Guadalajara, Road Ing. Ramón Padilla, Sánchez 2100, Las Agujas, Zapopan 45200, Jalisco, Mexico; lucia.barrientos@academicos.udg.mx; 6 Department of Entomology, Universidad Nacional Experimental Simón Rodríguez, Caracas 5130, Venezuela; hugocerda04@gmail.com

**Keywords:** essential oils, *Magnolia pugana*, chemical characterization, antioxidant activity, antibacterial activity, cytotoxic activity, bioautographic TLC

## Abstract

*Magnolia pugana* (Iltis & A. Vazquez) A. Vázquez & Carvajal, an endemic Mexican species of the Magnoliaceae family, has received limited phytochemical and pharmacological attention. This study reports, for the first time, the chemical composition and in vitro bioactivities of essential oils (EOs) obtained from its leaves, flowers, and seeds. EOs were analyzed by gas chromatography–mass spectrometry (GC-MS) and flame ionization detection (GC-FID), revealing cyclocolorenone, a sesquiterpene, as the major compound (38–40%) across all plant parts. Antioxidant activity was evaluated using DPPH• and ABTS• radical scavenging assays, complemented by bioautographic thin-layer chromatography (TLC). Antibacterial activity was determined by minimum inhibitory concentration (MIC) against human pathogenic bacteria, while cytotoxicity was assessed in MCF-7 (breast) and HT-29 (colon) cancer cell lines. Seed and flower EOs exhibited the highest antioxidant activity, with IC_50_ values of 21.5 mg/mL and 9.04 mg/mL, respectively. Strong antibacterial effects were observed against Gram-positive strains, particularly *Staphylococcus epidermidis* (MIC = 355.11 µg/mL) and *S. aureus* (MIC = 710.23 µg/mL). Leaf EO showed selective cytotoxicity toward MCF-7 cells (IC_50_ = 27.25 µg/mL), while seed EO was most active against HT-29 cells (IC_50_ = 54.01 µg/mL). These results suggest that *M. pugana* EOs, especially those from seeds, are a promising source of natural antioxidant, antimicrobial, and anticancer agents.

## 1. Introduction

The Magnoliaceae family, primarily found in montane cloud forests, is considered a representative group of angiosperms due to its ancestral traits and ecological importance [1]. Within this family, the genus *Magnolia* is taxonomically prominent and diverse, encompassing over 300 species globally [2]. Mexico, in particular, harbors approximately 11% of the world’s *Magnolia* species, positioning it as a center of diversity and endemism; however, many species are under threat due to anthropogenic activities [3].

Several *Magnolia* species have been historically valued for their medicinal uses. In Asia, *M. officinalis*, *M. obovata*, and *M. biondii* have been integral components of traditional Chinese and Japanese medicine, exhibiting various biological activities such as antioxidant, anti-inflammatory, antimicrobial, and anxiolytic effects [4]. On the American continent, the earliest described species was *M. dealbata*, locally known as “Eloxóchitl.” Historical accounts also describe the cultivation of *M. mexicana*, or “Yoloxóchitl,” in the royal medicinal gardens of Emperor Moctezuma, where it was prized for its cardiotonic properties and exquisite fragrance [5].

**Figure 1:**
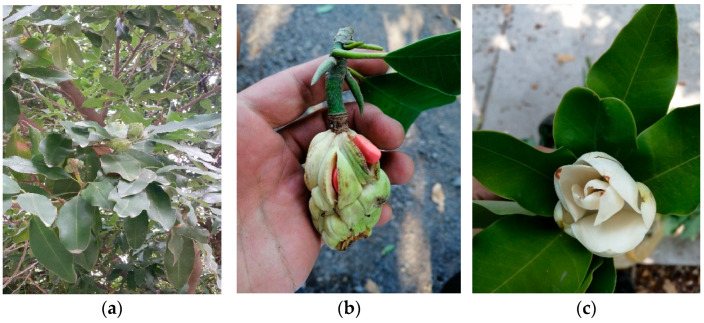
*Magnolia pugana*. (**a**) leaves, (**b**) fruit and seeds, (**c**) flowers.

Exploration efforts in central-western Mexico, particularly in the northern coastal and central zones of Jalisco, led to the discovery of morphological and ecological discontinuities between *Magnolia pacifica* subsp. *pacifica* and subsp. *pugana.* These differences justified the elevation of the latter to species level, as *Magnolia pugana* [6] (Figure 1). This species was named in honor of Luz María Villarreal de Puga, a distinguished botanist at the University of Guadalajara.

*M. pugana* is endemic to gallery forests of southern Zacatecas and central-northern Jalisco [7,8], where it is traditionally used for firewood and furniture. Its extremely restricted distribution (extent of occurrence: 2460 km^2^; area of occupancy: 114 km^2^) and fragmented populations have led to its classification as an endangered species by the Mexican government [9]. It exhibits a discontinuous flowering phase and a continuous fruiting period, with peaks in June and February, respectively [10]. This phenological behavior is essential for planning future conservation strategies and phytochemical sampling.

In light of increasing global interest in natural products, essential oils (EOs) from medicinal and endemic plant species have drawn significant attention. EOs are complex lipophilic mixtures of volatile compounds (mainly terpenes and phenylpropanoids) synthesized and stored in specialized structures such as secretory glands or trichomes [8,9,10]. These compounds are generally extracted by hydrodistillation, and their biological activity depends on the physical-chemical properties of both major (20–95%) and minor components, which may act synergistically or antagonistically [11,12,13].

EOs have demonstrated various biological properties, including antioxidant, anti-inflammatory, antimicrobial, antiviral, antimutagenic, and anticancer activities, which make them promising candidates for use in the pharmaceutical, cosmetic, and food industries [12,14]. However, such biological activity is highly influenced by factors such as plant part used, seasonality, genotype, and geographical location [15,16].

Despite its ecological importance and endangered status, *M. pugana* has not yet been phytochemically characterized, and its volatile profile remains unknown. The chemical composition of EOs often reflects taxonomic and environmental specificity, making them valuable for both chemotaxonomic and bioactivity studies [6]. Given the morphological differentiation of *M. pugana* from related species, as well as its distinct geographical distribution, this study addresses the urgent need for its biochemical profiling.

Therefore, this research provides the first comprehensive report on the chemical composition and biological activities of EOs extracted from the leaves, flowers, and seeds of *M. pugana*. The aim is to generate baseline data that supports the conservation and valorization of this endemic and threatened Mexican species.

## 2. Results and Discussion

### 2.1. Essential Oil Extraction

The main characteristics of essential oils extracted from *M. pugana* (MpEOs) are present in Table 1. In terms of yield, the seed extraction product showed the best percentage (~4.0%) but required double the time for hydrodistillation compared with leaves and flowers. In this case, it is important to mention that seeds were extracted manually from mature poly-follicles, and all the seeds were used, including the aril, in the distillation process, considering the seasonal maximum production peaks [17].

From a chemical, medicinal, and industrial point of view, plants represent a global interest due to the diversity of secondary metabolites they could generate. In this area, a wide exploration field is often linked to ethnopharmacy as the initial information point for research. Inside Jalisco are endemic populations of *Magnolia* genera; at this point, *M. pugana* was separated from *M. pacifica* based on the morphological attributes found [18]. *M. pugana* tree uses are associated with ornamental, furniture making, and fuel [19]. To our knowledge, there are no suitable studies about its essential oils.

As secondary metabolites, EOs undergo changes generally attributed to environmental conditions, soil factors, the species’ life cycle, and the extraction method employed [20]. In this case, this plant also evidenced seasonal development.

### 2.2. Chemical Analysis

**Figure 2:**
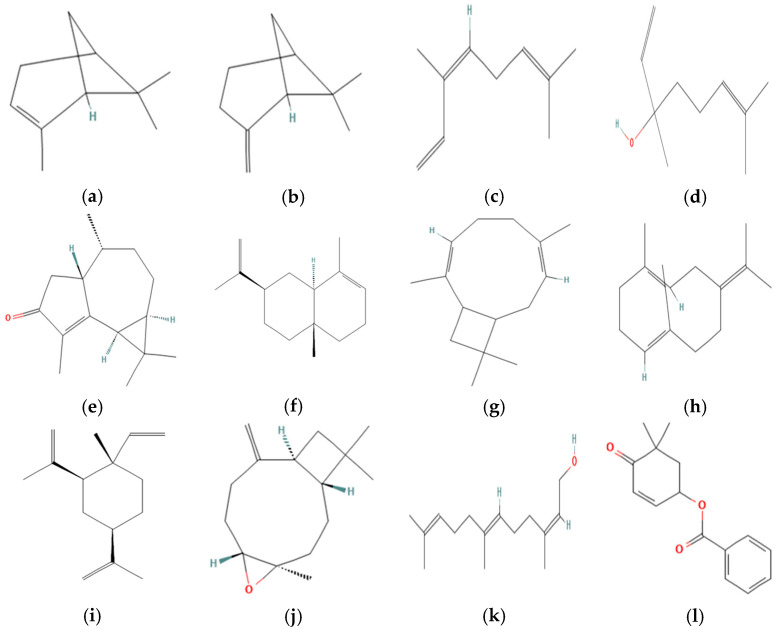
Chemical structures of the main constituents identified in the essential oils of *Magnolia pugana*. (**a**) α-Pinene; (**b**) β-Pinene; (**c**) (Z)-β-Ocimene; (**d**) Linalool; (**e**) Cyclocolorenone; (**f**) α-Selinene; (**g**) (I3)-Caryophylleine; (**h**) Germacrene B; (**i**) β-Elemene; (**j**) Caryophyllene oxide; (**k**) 2Z,6E-Farnesol; (**l**) Benzoic acid (5,5-dimethyl-4-oxo-2-cyclohexenyl)-ester. Structures obtained from PubChem (National Center for Biotechnology Information, 2025) [27].

The MpEO analysis carried out by GC-FID and GC-MS detected 58 compounds (See Supplementary Materials) and showed the presence of high concentrations of the oxygenated sesquiterpenes class (≅54.33%); among these, cyclocolorenone stands out as the main compound within 39–45%. The details of MPEO chemical compounds are summarized below for leaves (Table 2), seeds (Table 3), and flowers (Table 4). The chemical structures of the main components are illustrated in Figure 2.

In the case of *M. pugana* leaves, the main components were cyclocolorenone, β-pinene, linalool, α-pinene, and (Z)-β-ocimene. The presence of cyclocolorenone, i3-caryophylline, β-pinene, α-selinene, and B-germacrene is highlighted in seeds. Finally, in flowers, the composition showed cyclocolorenone, 2Z,6E-farnesol, benzoic acid (5,5-dimethyl-4-oxo-2-cyclohexenyl) ester, β-elemene, and caryophyllene oxide.

The GC-MS analysis showed that MpEOs are especially rich in cyclocolorenone, regardless of which part was used for the extraction. According to other studies, a wide variety of compounds have been identified, including farnesol and terpineol in *M. biondii* [19,20]; α- terpinene in *M. kwangsiensis* [12]; eucalyptol in *M. liliflora* [22]; and spathulenol in *M. ovata* [23]. In comparison with previous reports, our findings revealed no similarity in chemical composition; however, certain compounds may serve as taxonomic markers to differentiate species. Notably, cyclocolorenone, a tricyclic compound of the aromadendrane family with a bicyclo[5.3.0]decane skeleton [24], was identified for the first time in MpEOs. This compound is generally absent in most *Magnolia* species, with the exception of a floral EO from a southeastern U.S. population of *M. grandiflora*, where it was found up to 40%, along with other shared constituents such as β-pinene, β-elemene, and (2Z,6E)-farnesol [25]. In contrast, studies on other Mexican species such as *M. schiedeana* and *M. tamaulipana* have reported geranyl methyl ether as the dominant floral component [26], while preliminary analyses of *M. pacifica* and *M. vallartensis* from Jalisco indicated the absence of cyclocolorenone in their leaf and flower essential oils [3].

**Figure 3:**
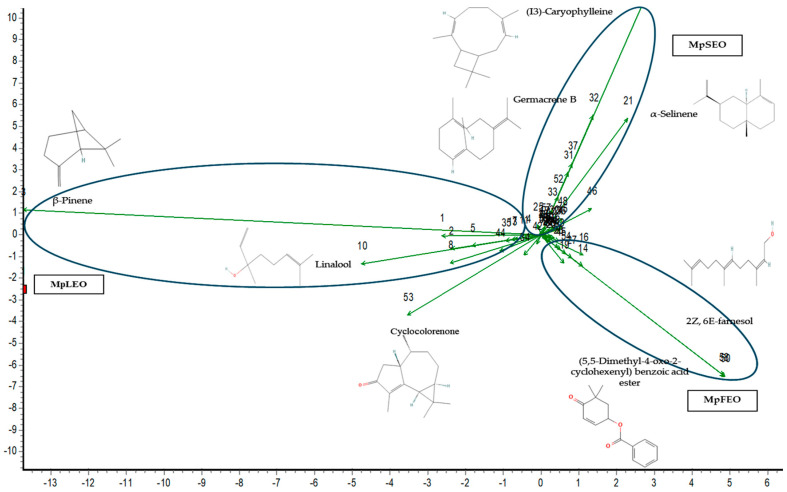
PCA plot of *M. pugana* essential oils. Component 1 (explained variation 60.67%); Component 2 (explained variation 39.33%). The numbers correspond to an identified chemical compound (1 to 58).

Based on the concentration of each compound, a principal component loading plot (Figure 3) was generated using the Community Analysis Package software Version 4.0 [28], with unstandardized input values. The analysis explained 60.67% of the variation along the x-axis and 39.33% along the y-axis. This analysis highlighted the compounds contributing most to the differentiation among essential oils, identified by the longest vectors. In leaf EO, key contributors were β-pinene, linalool, and cyclocolorenone; in seed EO: I3-caryophyllene, α-selinene, and germacrene B; and in flower EO: 2Z,6E-farnesol and the ester of (5,5-dimethyl-4-oxo-2-cyclohexenyl)-benzoic acid. As noted by [29], such multivariate analyses can reflect potential chemotaxonomic relationships in essential oil profiles, and their robustness increases when including additional species and populations.

### 2.3. Antioxidant Activity

The non-linear regressions of the curves obtained by comparing the percentages of inhibition and the concentrations of MpEOs, allowed us to determine the quantification of the antioxidant activity expressed in the IC_50_ values (mg/mL), which are summarized in Table 5. The values closest to zero correspond to the tested agents representing the highest antioxidant activity.

As expected, since it is a synthetic compound, the positive control BHT showed the lowest values in the two assays: IC_50_ DPPH (1.66 mg/mL) and IC_50_ ABTS (0.24 mg/mL). Among the MpEOs (Figure 3), first comparing DPPH radical, the lowest IC_50_ value corresponded to MpSEO (21.5 mg/mL), followed by MpFEO (27.9 mg/mL) and MpLEO (37.4 mg/mL). In the case of the ABTS radical, the following behaviors were obtained with the MpEOs: MpFEO showed the lowest IC_50_ value (9.04 mg/mL), followed by MpLEO (14.8 mg/mL) and MpSEO (17.4 mg/mL).

The IC_50_ values obtained in this study also suggest an affinity between the chemical nature of the compounds and the type of radical evaluated. DPPH is a lipophilic radical that reacts mainly with hydrophobic antioxidant compounds, while ABTS•+ is hydrophilic and can react with both lipophilic and hydrophilic molecules [30]. In this context, a study on the floral EO of *M. biondii* using the “electronic nose” (E-nose) technique observed variations in the scavenging activity of individual components against DPPH and ABTS radicals; although the composition of *M. biondii* is characterized by monoterpenes, it was found, for example, that α-pinene (C_10_H_16_) scavenged 5.9% of DPPH compared to 23.6% of ABTS [31]. These differences emphasize the importance of applying multiple assays to obtain a comprehensive evaluation of antioxidant capacity.

Regarding antioxidant activity, it is often associated with the presence of phenolic compounds and other secondary metabolites containing conjugated double bonds, which generally display stronger antioxidant effects [32]. In line with this, another study demonstrated that the seed EO of *M. grandiflora* exhibits remarkable radical scavenging properties; in an in vivo assay, it was confirmed that some of its bioactive components act as primary and secondary antioxidants by neutralizing free radicals and inhibiting lipid peroxidation [33]. Similarly, in the MpEOs, although IC_50_ values were higher compared to the synthetic antioxidant BHT—as expected for complex natural mixtures—they nevertheless showed significant radical scavenging capacity. MpSEO displayed the strongest activity in the DPPH assay, whereas MpFEO was more active in the ABTS assay.

#### Antioxidant Bioautography

**Figure 4:**
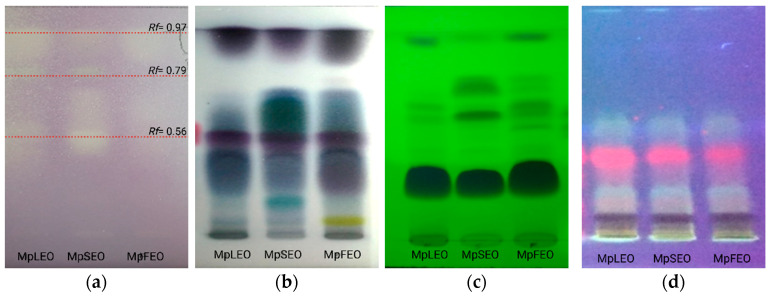
MpEOs developed on HPTLC plates. (**a**) Bioautographic antioxidant activity with DPPH solution, (**b**) with vanillin-sulfuric acid, (**c**) Fluorescence at 254 nm, (**d**) Fluorescence at 366 nm. MpLEO (leaves), MpSEO (seeds), MpFEO (flowers), Rf (retention factor). Volume: 20 µL of essential oils diluted in ethanol.

The separation of the MpEOs by thin-layer chromatography and subsequent development with the DPPH radical showed the presence of three active fractions (Figure 4), which may be directly related to the antioxidant activity previously described.

The antioxidant-active fractions were analyzed by GC–MS, which allowed the detection of 20 related compounds. As presented in Table 6, fractions f1 (*Rf* = 0.97) and f2 (*Rf* = 0.79) appeared consistently across the three oils. Key constituents such as β-elemene (3.5% in MpFEO), E-caryophyllene, germacrene D, (E)-γ-bisabolene, caryophyllene oxide, and τ-muurolol are closely linked to the antioxidant activity observed.

### 2.4. Antibacterial Activity

The results of the minimum inhibitory concentration (MIC) assay against the seven human pathogenic bacteria are presented in Table 7. The MIC values showed that Gram-negative bacteria were generally less sensitive to the MpEOs, with *E. coli* being the most resistant (22.7 mg/mL), while *K. pneumoniae* was comparatively more susceptible (2.84 mg/mL for MpSEO). This trend reflects the protective role of the outer membrane in Gram-negatives, although differences in mobility and capsule production may influence susceptibility, as *K. pneumoniae* is non-motile but encapsulated [34]. In contrast, Gram-positives exhibited greater sensitivity, with *S. aureus* and especially *S. epidermidis* showing the lowest MIC values (0.71 and 0.35 mg/mL, respectively), consistent with their non-sporulating and immobile nature [35]. Conversely, *B. subtilis* was less affected (11.36 mg/mL), which may be related to its ability to form endospores and its motility [36], conferring increased tolerance.

Essential oils, have emerged as promising antimicrobial agents due to their diverse mechanisms of action, including disruption of cell membranes, inhibition of protein synthesis, and interference with DNA replication [37]. This findings demonstrate MpEOS could act over pathogens considered responsible for half of the global deaths caused by bacterial nosocomial infections, such as *S. aureus, E. coli*, and *K. pneumoniae* [38].

Overall, MpSEO displayed the strongest activity, likely due to its higher content of oxygenated sesquiterpenes such as cyclocolorenone. While not as extensively researched as some other antibacterial agents, there is evidence suggesting its potential in combating bacterial infections [39,40]. Cyclocolorenone, belongs to the tricyclic-aromadendrene-type sesquiterpenes and are widely distributed and exhibit a range of biological activities, including antibacterial properties [41]. This compound was isolated from *Solidago canadensis* [42], *Solidago virgaurea* [43], *Critonia aromatisans* [44] and in *Magnolia grandiflora* [45]. For example, in *S. virgaurea* the essential oil contains 29.5% of cyclocolorenone and antibacterial results were similar compared with MpEOs. These results highlight both the structural and physiological traits of the bacteria and the chemical composition of the oils as key factors in determining antimicrobial efficacy, underscoring the potential of MpEOs as natural antimicrobial agents with possible food, cosmetic, and biomedical applications.

#### Antibacterial Bioautography

**Figure 5:**
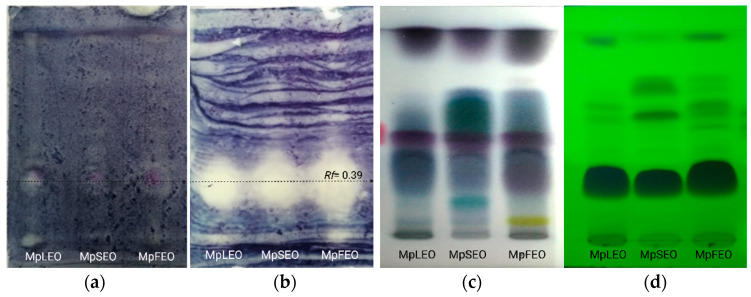
MpEOs antibacterial activity developed by TLC-DB. (**a**) *E. coli* ATCC^®^ 8739, (**b**) *S. aureus* ATCC^®^ 6538p, (**c**) Plate with vanillin-sulfuric acid, (**d**) Plate under fluorescence at 254 nm. MpLEO (leaves), MpSEO (seeds), MpFEO (flowers), *Rf* (retention factor). Volume: 20 µL of essential oils diluted in ethanol.

As a complementary approach to the antibacterial assays, direct bioautographic tests on TLC plates allowed visualization of specific fractions corresponding to compounds interacting with bacterial growth. Distinct responses were observed between Gram-negative and Gram-positive bacteria, consistent with the results of the broth microdilution assay. Accordingly, Figure 5 displays the inhibition pattern against *E. coli* ATCC^®^ 8739 and *S. aureus* ATCC^®^ 6538p. Visual inspection revealed a marked difference in antibacterial activity between bacteria, particularly in relation to the fraction (*Rf:* 0.39), which was weakly active against *E. coli* but clearly visible and more extensive against *S. aureus*. This fraction was consistently present in the leaf, seed, and flower EOs of *M. pugana*.

These differences are largely attributed to structural distinctions between Gram-positive and Gram-negative bacteria. As noted by [46], Gram-negative bacteria possess an outer membrane rich in lipopolysaccharides, making them more resistant to antibiotics. However, ref. [47] emphasized that antibacterial activity also depends on the chemical composition of the essential oil and the molecular affinity of its constituents to bacterial membranes.

### 2.5. Cytotoxic Activity

**Figure 6:**
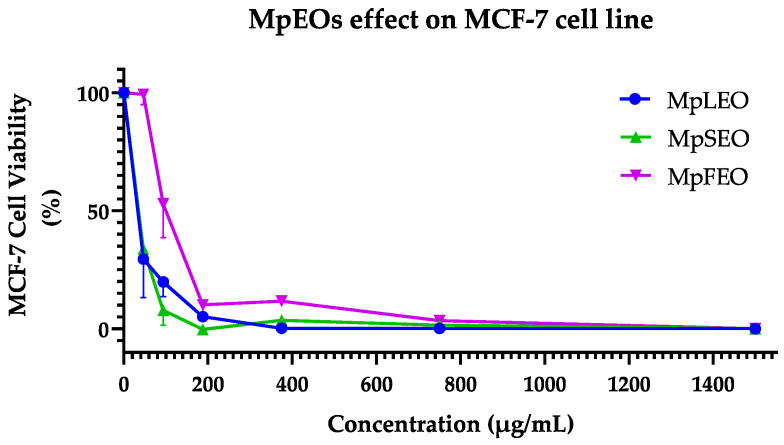
MpEOs and cyclophosphamide cytotoxicity on MCF-7 (breast adenocarcinoma) cell line. Cell viability of MCF-7 expressed as a percentage (vertical axes) versus concentrations (µg/mL) of EO (horizontal axes) after 48 h of contact. R^2^: Coefficient of determination in non-linear dose–response curves.

The MpEOs showed different behaviors in the cytotoxicity assays (Table 8). As a first point, in the case of the MCF-7 cell line, as seen in Figure 6, the curves obtained when confronting the concentrations of MpEOs and percentage of viability presented a dose–response type behavior. In the case of MpSEO and MpLEO, with the first tested concentration of 46.88 µg/mL, a decrease between 50 and 25% of the viability could be evidenced, reaching a maximum efficacy (values close to 0% of viability) between 375 and 1500 µg/mL. Unlike MpFEO, which required a higher concentration of 93.75 µg/mL to reach a decrease within (75–50%) and reach a maximum efficacy between 750 and 1500 µg/mL. On the other hand, the control with cyclophosphamide at 125 µg/mL managed to reduce a viability percentage of less than 25% and achieve its maximum effect from 250 to 1000 µg/mL.

**Figure 7:**
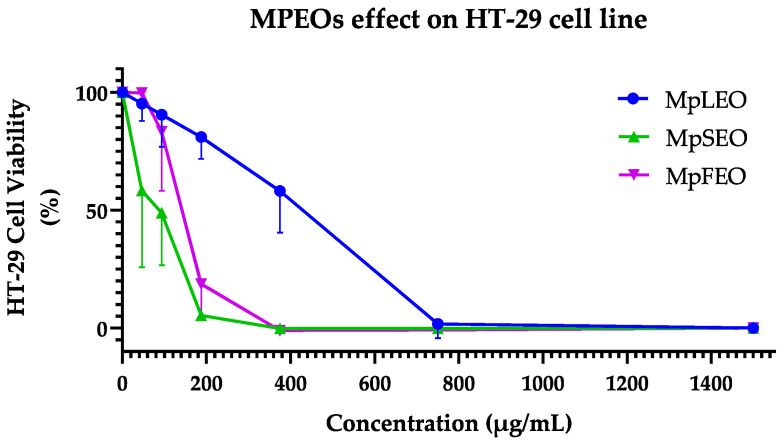
MpEOs and cyclophosphamide cytotoxicity on HT-29 (colorectal adenocarcinoma) cell line. Cell viability of HT-29 expressed as a percentage (vertical axes) versus concentrations (µg/mL) of EO (horizontal axes) after 48 h of contact. R^2^: Coefficient of determination in non-linear dose–response curves.

For the HT-29 cell line, as shown in Figure 7, the concentration curves of MpEOs and control versus the percentage of cell viability also showed a dose–response behavior. In the case of MpSEO, with the first tested concentration of 46.88 µg/mL, an average decrease of 50% in viability could be observed, and maximum efficacy was reached from 187.5 to 1500 µg/mL. MpFEO, on the other hand, required a higher concentration of 187.5 µg/mL to reach a value lower than 25% of viability, reaching the maximum efficiency between 375 and 1500 µg/mL. In contrast, MpLEO showed a weaker cytotoxic effect; on average, from 375 µg/mL, it decreased to 50% viability, and its maximum efficacy was from 750 to 1500 µg/mL. On the other hand, Cyclophosphamide achieved a viability reduction of less than 25% at 500 µg/mL and did not reach a maximum effect even at 1000 µg/mL.

This study represents the first report on the cytotoxic effects of *Magnolia pugana* essential oils (EOs) against the MCF-7 (breast cancer) and HT-29 (colon cancer) cell lines. Although the cytotoxic activity of each individual component was not specifically evaluated, chemical profiling revealed a high abundance of oxygenated sesquiterpenes, particularly cyclocolorenone (C_15_H_22_O), which was the predominant constituent (39–45%) across all oils. This sesquiterpene has been previously associated with selective anticancer activity. For instance, ref. [48] reported that cyclocolorenone, present at 25% in the EO of *Montanoa guatemalensis* (Asteraceae), contributed to its cytotoxic effects against the MDA-MB231 breast cancer cell line. Similarly, ref. [49] identified 18% cyclocolorenone in the EO of *Drymis brasiliensis* (Winteraceae), which significantly reduced cell proliferation in U-138MG (glioblastoma, 42.5%) and T24 (bladder carcinoma, 67.8%) cells, with late apoptosis as the suggested mechanism.

Based on the IC_50_ values obtained in this study, the three EOs from *M. pugana* displayed a selective response toward MCF-7 cells. The leaf EO exhibited the most pronounced selectivity, whereas cyclophosphamide, used as a reference drug, showed no selectivity between MCF-7 and HT-29. According to the classification system proposed by ref. [50], which defines cytotoxic activity as strong (IC_50_ < 50 µg/mL), moderate (50–200 µg/mL), low (200–1000 µg/mL), or inactive (IC_50_ > 1000 µg/mL), the oils can be categorized as follows: Leaf EO—strong cytotoxicity against MCF-7 and low activity against HT-29; Seed EO—strong activity on MCF-7 and moderate activity on HT-29; and Flower EO—moderate cytotoxic effects on both cell lines.

The cytotoxicity assays highlighted constitutive differences between the cell lines. MCF-7, a hormone-dependent breast cancer model, expresses functional estrogen receptors and shows a pleiotropic response to estrogen stimulation, promoting proliferation in vitro [51]. In contrast, HT-29 is a pluripotent intestinal cancer cell line used to study both cancer and cell differentiation, characterized by tight junctions and apical brush-border structures containing villin, a protein typical of intestinal microvilli [52]. As noted, such specific traits may explain why a phytocomplex like an essential oil shows strong activity in one line but remains inactive in another, reflecting differences in the molecular target networks—including receptors, second messengers, and DNA expression regulators—between cell lines [53].

These findings highlight the potential of MpEOs, particularly the leaf and seed oils, as promising candidates for further exploration in cancer-related applications. The antiproliferative effects of EOs are believed to be associated with their chemical composition and physicochemical properties. Although the precise mechanisms remain to be elucidated, previous studies have reported that EOs can trigger apoptosis and DNA fragmentation through activation of caspases 3 and 9 [54], or induce necrosis [55]. Due to their lipophilic nature and structural diversity, EOs are capable of traversing the cell wall and cytoplasmic membrane, disrupting polysaccharide, fatty acid, and phospholipid layers. This permeabilization leads to membrane depolarization, altered calcium ion flux, and ultimately to apoptotic or necrotic cell death [56].

## 3. Materials and Methods

### 3.1. Plant Material

The delimited study area comprised two rural sites of private access, located 8–10 km west of Tesistán, municipality of Zapopan, state of Jalisco (20°49′11.71″ N, 103°34′42.37″ O). *M. pugana* plant material samples were randomly collected from 12 individuals, with approximately 1 kg of plant parts (leaves, flowers, and seeds). Phenophases were also considered for plant collection: seeds (February), flowers (May–June), and leaves (November). A pole was used to facilitate the collection of samples that were difficult to reach in the aerial parts of the trees. The plant material was stored in plastic containers to protect it from light and heat until it was transferred to the laboratory. At the Biotechnology Laboratory of the Department of Botany and Zoology, Universidad de Guadalajara-Mexico, the samples were cleaned and frozen at −20 °C until distillation.

### 3.2. Extraction of Essential Oils

The *M. pugana* essential oils (MpEOs) were extracted in the Extractables Laboratory, Department of Pulp and Paper, Universidad de Guadalajara-Mexico, by hydrodistillation using a Clevenger-type apparatus. A 2.0 L capacity glass distillation with water recirculation and a cooling system was used for the procedure at 100 °C. For the process, 150 g of fresh plant parts were used to preserve the delicate aromatic compounds, especially in flowers. The extraction time employed was for leaves (4 h), seeds (8 h), and flowers (4 h).

### 3.3. Chemical Composition of Essential Oils

The MpEOs were analyzed by GC-MS using the method employed by [57,58]. Individual samples were prepared by diluting 10 µL of each EO in 990 µL dichloromethane (Merck, Darmstadt, Germany). The injection volume was 2 µL. The chemical composition was determined in the Life Sciences Laboratory of the Universidad Politécnica Salesiana-Ecuador, in a Bruker GC-MS system (Bruker Daltonics, Fahrenheitstraße, Bremen, Germany) chromatograph model 436 SCION^®^ coupled to a triple quadrupole mass detector model TQ-EVOQ. A Bruker BR-5ms column (5% phenyl, 95% dimethylpolysiloxane), 30 m length × 0.25 mm internal diameter, 0.25 µm film thickness, maximum analysis temperature 350 °C, and an Agilent DBWax column with polyethylene glycol (Agilent, Santa Clara, CA, USA) with a length of 20 m, a thickness of 0.10 mm, and a film thickness of 0.20 were used. The carrier gas was helium (99.999%) at a flow rate of 1 mL/min with a split ratio of 1:50. The temperature program used for the analysis was 50 °C (1 °C/min for 50 min) until 100 °C, then 5 °C/min for 30 min until reaching 250 °C, and finally 10 min at 250 °C.

Mass spectrometer conditions were ionization energy: 70 eV, emission current: 10 µAmp, scan rate: 1 scan/s, mass range: 35–400 Da, trap temperature: 220 °C, transfer line temperature: 260 °C. The compounds were identified by comparison of mass spectra using the WILEY-NIST 2014 [21] and Adams 2017 [11] libraries for essential oil compounds. The software used in this compound analysis was MS-Workstation (Bruker Daltonics, Fahrenheitstraße, Bremen, Germany), which integrated with NIST-MS-Search.

In addition, experimental arithmetic retention index (AI) values were calculated using the van den Dool and Kratz Equation (1) [59], based on the retention times of a homologous series of n-alkanes analyzed under the same chromatographic conditions, according to the formula:

(1) $${AI}_{x}=\left(100\;\times \;C_{a}\right)\;+\left\{100\;\times \frac{{RT}_{x}-{RT}_{a}}{{RT}_{A}-{RT}_{a}}\right\},$$

where ${AI}_{x}$ is the arithmetic retention index of a compound of interest, $C_{a}$ is the number of carbon atoms of the n-alkane eluting immediately before the compound, ${RT}_{x}$ is the retention time of the compound of interest, ${RT}_{a}$ is the retention time of the preceding n-alkane, and ${RT}_{A}$ is the retention time of the subsequent n-alkane.

### 3.4. Antioxidant Activity

Two assays were used for the quantification of antioxidant activity in microplates: the 2,2-diphenyl-1-picrylhydrazyl radical (DPPH−) uptake assay proposed by ref. [60] and the 2,2′-azino-bis-3-ethylbenzothiazoline-6-sulphonic acid (ABTS−+) reduction assay indicated by ref. [61]. These assays were conducted at the Toxic Residues Laboratory, Department of Public Health, Universidad de Guadalajara-Mexico. For both assays, butylated hydroxytoluene (BHT; Merck, Darmstadt, Germany) was used as a synthetic antioxidant control, and methanol (Merck, Darmstadt, Germany) as a negative control.

For the DPPH• assay, the oils and controls were diluted in methanol to obtain six test concentrations: MpEOs (1420.45–45,454.54 µg/mL) and BHT (340.91–5454.54 µg/mL). A methanolic solution of DPPH• (0.2 mg/mL; Merck, Darmstadt, Germany) was prepared, and once the radical was dissolved, its absorbance was verified to be between 0.9 and 1.1 at 517 nm. The solution was then stored in an amber bottle and refrigerated until use. In a 96-well microplate, 20 µL of MpEOs solutions, BHT solutions, and methanol were placed in separated wells. Subsequently, 80 µL of the DPPH• solution was added. Three wells containing 100 µL of methanol served as blanks. The plate was manually shaken for 5 min and left to stand in the dark at room temperature for 30 min. Finally, the absorbance was measured at 517 nm using a Biotek ELX800 reader (Biotek Instruments, Winooski, VT, USA).

For the ABTS•+ assay, 100 mL of an aqueous solution of ABTS (3.84 mg/mL; Merck, Darmstadt, Germany) was prepared (Solution A). In parallel, 100 mL of an aqueous solution of potassium persulfate (K_2_S_2_O_8_; 37.84 mg/mL) was prepared (Solution B). The ABTS•+ radical cation was then generated by adding 17.6 µL of Solution B to each milliliter of Solution A. This mixture was stored in an amber flask, protected from light, at room temperature for 16 h. Finally, the solution was diluted with methanol (Merck, Darmstadt, Germany) until an absorbance of 0.8–1.0 at 734 nm was reached, as verified on a Jenway 6405 UV/Vis spectrophotometer (Cole-Parmer, Vernon Hills, IL, USA). Solutions of MpEOs (375.00–6000.00 µg/mL) and BHT (250.00–4000.00 µg/mL) were prepared. In a 96-well microplate, 20 µL of MpEOs solutions, BHT solutions and methanol were pipetted into separate wells. Subsequently, 280 µL of the ABTS•+ solution was added to the wells. Additionally, three wells containing 300 µL of methanol served as blanks. The microplate was manually shaken for 5 min and kept in the dark for 20 min. At the end of this period, the absorbance was measured at 734 nm using a Biotek ELX800 reader (Biotek Instruments, Winooski, VT, USA).

After these analyses, the respective percentages of inhibition of the radicals were determined using the formula below.

(2) $$\%\;I\;\left(\mathrm{DPPH}\;/\;\mathrm{ABTS}\right)=\left(1-\left(\frac{{A\mathrm{bs}}_{\mathrm{Spl}}-{A\mathrm{bs}}_{\mathrm{Bl}}}{{A\mathrm{bs}}_{\mathrm{Ctrl}}-{A\mathrm{bs}}_{\mathrm{Bl}}}\right)\right)\times 100,$$

where %I is the percentage of inhibition of the DPPH− or ABTS−+ radicals, A_Bl_ is the absorbance of the blank, A_Spl_ is the absorbance of the sample and A_Ctrl_ is the absorbance of the negative control. The 50% Inhibitory Concentrations (IC_50_) were determined by regression between the concentrations tested and the inhibition percentages obtained for each sample, and the value was expressed in mg/mL.

#### Autobiographic Antioxidant Activity

The assay followed the protocol described by [57]. Two HPTLC silica gel 60 F_254_ plates (Merck, Darmstadt, Germany) were activated at 105 °C in a conventional oven. MpEOs ethanolic solutions (60 mg/mL) were prepared, and 12 μL were applied to each plate using a 100 μL microsyringe (Hamilton Company, Reno, NV, USA) with 1 cm spacing between spots. Plates were developed in a glass chamber pre-saturated with the mobile phase consisting of toluene/ethyl acetate/petroleum ether (93:7:20, *v*/*v*/*v*). After elution, plates were air-dried for two hours at room temperature to allow solvent evaporation.

The first plate was observed under UV light to visualize the separation of total MpEOs fractions. Subsequently, the same plate was sprayed with a methanolic DPPH solution (20 mg/mL) and allowed to dry at ambient conditions. Antioxidant-active bands appeared as clear zones against a purple background, indicating free radical scavenging activity. The retention factor (Rf) for each band was calculated according to the following formula:

(3) $$\mathrm{Rf}\;=\frac{D_{i}\;(\mathrm{cm})}{D_{s}\;(\mathrm{cm})},$$

where *Rf* is the retention factor; Di is the distance traveled by the band of interest from the origin; and Ds is the distance traveled by the solvent front from the origin.

On the second eluted plate, these fractions were marked in correspondence with the Rf values of the first plate. Subsequently, the silica was carefully scraped, and the collected material was stored in an amber vial. The sample was then dissolved in 2 mL of dichloromethane (Merck, Darmstadt, Germany), filtered through 0.45 μm PVDF membranes, and injected into the GC–MS system under the same analysis and identification parameters described in Section 3.3.

### 3.5. Antibacterial Activity

This activity was assessed in the Food Safety Laboratory, Department of Public Health, Universidad de Guadalajara-Mexico, following the broth microdilution method described by [62,63], with a modification proposed by [57] in which 2,3,5-triphenyltetrazolium chloride (TTC; Merck, Darmstadt, Germany) was used as a visual indicator of bacterial cell viability.

Seven American Type Culture Collection (ATCC; Manassas, VA, USA) human pathogenic bacterial strains were tested: Gram-negative (*E. coli* 25922, *E. coli* 8739, *K. pneumoniae* 10031, *S. typhimurium* 14028) and Gram-positive (*S. aureus* 6538p, *S. epidermidis* 14990, *B. subtilis* subsp. spizizenii 6633). Colonies were subsequently isolated on Trypticase Soy Agar (TSA; Becton, Dickinson and Company, Franklin Lakes, NJ, USA) and stored on slants.

For the determination of the minimum inhibitory concentration (MIC), bacterial suspensions were adjusted to the 0.5 McFarland turbidity standard (~10^8^ CFU/mL), then diluted to 10^7^ CFU/mL. Stock solutions of the oils were prepared by dissolving 210 mg of each in a mixture of 50% ethanol and 2% Tween^®^ 20 (Merck, Darmstadt, Germany), which facilitated solubilization of the hydrophobic compounds. At these concentrations, ethanol and Tween^®^ 20 do not exhibit antimicrobial activity, as verified by the negative control. Ampicillin (43.1 mg/mL; Merck, Darmstadt, Germany) was used as the positive control, while the negative control contained 125 μL/mL of saline solution with 50% ethanol and 2% Tween^®^ 20.

MIC assays were carried out in 96-well microplates. Each well was filled with 100 μL of Mueller–Hinton Broth (MHB; Becton, Dickinson and Company, Franklin Lakes, NJ, USA). Then, 100 μL of each stock solution was added to wells A1–H1 and serially diluted across 11 columns using a multichannel pipette. Except for the sterility control, all wells were inoculated with 10 μL of bacterial suspension (10^7^ CFU/mL) to reach a final concentration of 10^5^ CFU per well. Plates were incubated at 35 ± 2 °C for 24 h (48 h for *S. aureus* and *S. epidermidis*). After incubation, 20 μL of aqueous TTC solution (20 mg/mL) was added to each well, followed by an additional 30 min incubation at 35 ± 2 °C. The minimum inhibitory concentration (MIC) was defined as the lowest concentration of the tested agent showing no visible bacterial growth after incubation, with TTC used only as a color indicator to facilitate visual assessment.

#### Autographic Antibacterial Activity

The assay followed the Thin Layer Chromatography-Direct Bioautography (TLC-DB) by immersion method described by [64], with modifications. TLC silica gel 60 F_254_ polymer, 10 × 10 cm plates (Merck, Darmstadt, Germany) were preconditioned at 45 °C for 12 h. Bacterial strains: *E. coli* ATCC^®^ 8739 and *S. aureus* ATCC^®^ 6538p, were first grown on Muller Hinton Agar (MHA; Becton, Dickinson and Company, Franklin Lakes, NJ, USA), then transferred to 100 mL of Nutrient Broth (Becton, Dickinson and Company, Franklin Lakes, NJ, USA) supplemented with 0.5% agar, and incubated at 37 °C—overnight for *E. coli*, and 48 h for *S. aureus*. Cultures were adjusted to an absorbance of 0.4 at 600 nm in a Shimadzu UVmini-1240 spectrophotometer (Shimadzu, Kyoto, Japan) corresponding to ~4 × 10^7^ CFU/mL. Once at room temperature, TLC plates were marked with graphite and spotted with 10 μL of MpEOs methanolic solutions (30 μL/mL) in horizontal bands (10 mm wide, 1 cm from the base) using a 50 μL microsyringe (Hamilton Company, Reno, NV, USA). Plates were developed in a chromatographic chamber saturated with the mobile phase (toluene/ethyl acetate/petroleum ether, 93:7:20, *v*/*v*/*v*), then dried with cold air for ~60 min, fluorescent bands were observed at 254 nm to verify compound separation. The assay was performed in triplicate. Finally, plates were placed in a humid chamber at 25 °C for 90 min to promote bacterial growth. After incubation in the humid chamber, the TLC plates were carefully immersed for 10 s in 50 mL of standardized bacterial suspension placed in a 20 cm Petri dish, then air-dried for 2 min. Plates were incubated at 37 °C for 24 h. Following incubation, the plates were submerged for 60 s in 50 mL of an aqueous 3-(4,5-dimethylthiazol-2-yl)-2,5-diphenyltetrazolium bromide (MTT; Merck, Darmstadt, Germany) solution (0.6 mg/mL) containing 0.1% Triton X-100 (Merck, Darmstadt, Germany), then incubated again for 1 h at 37 °C. To stop the reaction, plates were briefly immersed in 70% ethanol for 10 s and air-dried for 5 min. Antibacterial activity was visualized as clear zones or spots against a bluish background. The Rf values of active fractions were calculated by measuring migration distances.

### 3.6. Cytotoxic Activity

Cytotoxic activity was assessed in vitro using the MTT assay as a viability indicator. The assay was conducted on two adherent human cancer cell lines: MCF-7 (ATCC^®^ HTB-22™, breast adenocarcinoma) and HT-29 (ATCC^®^ HTB-38™, colorectal adenocarcinoma). Cell lines were provided by the Institute for Biomedical Research and Molecular Genetics, Department of Molecular and Cell Biology, Universidad de Guadalajara-Mexico.

Cells were cultured and preconditioned for two weeks under standard conditions (35 °C, 5% CO_2_, 95% humidity). MCF-7 cells were maintained in Dulbecco’s Modified Eagle Medium (DMEM; Merck, Darmstadt, Germany) supplemented with 10% fetal bovine serum (FBS; Merck, Darmstadt, Germany), 1% glucose, and antibiotics (streptomycin/penicillin, Merck, Darmstadt, Germany); HT-29 cells were cultured in McCoy’s 5A (Merck, Darmstadt, Germany) medium with similar supplementation. Once cultures reached 70–80% confluence, as confirmed by phase-contrast microscopy (Olympus Optical, CK40, Tokyo, Japan), cells were detached using 4 mL of 0.25% trypsin-EDTA solution (Merck, Darmstadt, Germany) and incubated for 10 min at 35 °C. Cells were washed twice with sterile culture medium by centrifugation (10 min at 1500 rpm), and the pellet was resuspended in 1 mL of sterile medium. Cell density was estimated by mixing 10 μL of the cell suspension with 20 μL of medium and 20 μL of Trypan Blue solution (Merck, Darmstadt, Germany). Live cells were counted using a Neubauer chamber under. The final dilution was adjusted to seed approximately 15,000 cells per well.

The cytotoxicity assay was conducted in flat-bottom 96-well microplates following the internal protocol of the Institute for Biomedical Research and Molecular Genetics, adapted from the original method of [65]. Inside a laminar flow hood, 200 μL of the prepared cell suspension was pipetted into each well, except for the last three columns, which served as blanks. Plates were incubated for 24 h at 35 °C, 5% CO_2_, and 95% humidity. Test solutions of MpEOs (46.88–1500 µg/mL) and cyclophosphamide as a positive control (125–1000 µg/mL, Merck, Darmstadt, Germany) were prepared. Then, 50 μL of each concentration was added to the corresponding wells and incubated for 48 h under the same conditions. After incubation, wells were carefully emptied, and contents were discarded into a container with 5% NaClO. Next, 100 μL of MTT solution (5 mg/mL in culture medium) was added to each well, and plates were incubated for 4 h. After removing the MTT solution, 100 μL of DMSO (Merck, Darmstadt, Germany) was added to dissolve the formazan crystals. Plates were gently agitated for 1 min, and absorbance was measured at 595 nm using a Multiskan Ascent microplate reader with Ascent Software v2.6 (Thermo Scientific, Waltham, MA, USA).

Cell viability percentages were calculated based on absorbance values using the following formula:

(4) $$\%\mathrm{CV}=\left(\frac{{\mathrm{Abs}}_{\mathrm{Spl}}-{\mathrm{Abs}}_{\mathrm{Bl}}}{{\mathrm{Abs}}_{\mathrm{Ctrl}}-{\mathrm{Abs}}_{\mathrm{Bl}}}\right)\times 100$$

where %CV is the percentage of cell viability, AbsS_pl_ is the absorbance of treated cells, AbsC_trl_ is the absorbance of untreated control cells, and AbsB_l_ is the absorbance of the blank. The 50% inhibitory concentrations (IC_50_) were calculated by plotting the tested concentrations against the corresponding inhibition percentages for each EO and the BHT control. IC_50_ values were expressed in mg/mL.

### 3.7. Statistical Analysis

All experiments were performed in triplicate. Raw data were processed using Microsoft™ Excel 2023 for Windows^®^ (Microsoft Corporation, Redmond, WA, USA). Chemical composition of the essential oils (EOs) was qualitatively analyzed using the Jaccard similarity index with CAP software (Community Analysis Package v1.52, Pisces Conservation Ltd., Pennington, Lymington, UK). Based on the relative composition percentages, a Kruskal–Wallis ANOVA was conducted, followed by Dunn’s multiple comparisons test to determine significant differences. IC_50_ values for antioxidant and cytotoxic activities were calculated through logarithmic regression of dose–response curves (concentration vs. inhibition percentage) using GraphPad Prism v8.0.2 for Windows^®^ (GraphPad Software, San Diego, CA, USA; www.graphpad.com), with 95% confidence intervals. These IC_50_ values, along with MIC values from the antibacterial assays, were subjected to one-way ANOVA to assess significance. Post hoc comparisons were performed using Tukey’s test (for antioxidant and cytotoxic activity) and Fisher’s LSD test (for antibacterial activity), with a significance level of α = 0.05.

## 4. Conclusions

This study reports, for the first time, the chemical composition of leaf, seed, and flower oils of *Magnolia pugana* obtained by hydrodistillation and analyzed by GC/MS. The oils were found to be rich in oxygenated sesquiterpenes, with cyclocolorenone as the predominant compound. Although quantitative differences among plant parts were minor, qualitative variations revealed a moderate similarity between seeds and flowers.

In vitro assays demonstrated relevant biological activities, with antioxidant effects depending on the plant organ, stronger antibacterial responses against Gram-positive bacteria, and promising cytotoxic activity in human cancer cell lines. These findings suggest that *M. pugana* represents a valuable natural source of sesquiterpenes with potential biomedical applications. Further multidisciplinary research is recommended to deepen the understanding of its pharmacological properties and therapeutic prospects.

## Figures and Tables

**Table 1:** *M. pugana* essential oils characteristics.

Plant Part	Weight (g)	EO (mL)	Yield	Density	RI	Odor	Color
Leaves ^1^	150	≅0.49	0.33 ± 0.3%	0.923	1.505	Pungent, woody, bitter	Pale yellow
Flowers ^1^		≅0.42	0.28 ± 0.4%	0.962	1.510	Sweet, citric	Light yellow
Seeds ^2^		≅6.00	4.00 ± 0.2%	0.956	1.509	Sweet, woody	Light yellow

Note: Hydrodistillation time: ^1^ 4 h, ^2^ 8 h extraction. EO: Essential oil. RI: Refraction index.

**Table 2:** Chemical composition of *Magnolia pugana* leaves essential oil (MpLEO) analyzed by GC/MS.

No.	Compound	Apolar DB-5 Column		DB-Wax Column		Total (%)	S.D.
		AI ^Teo^	AI ^Exp^	AI ^Teo^	AI ^Exp^		
**1**	**α-pinene**	932	928	1025	1022	**3.73**	±1.50
**2**	Camphene	946	943	1068	1060	2.87	±0.83
**3**	**β-pinene**	974	972	1110	1107	**20.45**	±4.00
**4**	Myrcene	988	985	1160	1157	0.41	±0.06
**5**	δ-3-carene	1008	1005	1147	1140	2.20	±0.32
**6**	Limonene	1024	1020	1198	1195	0.85	±0.03
**7**	**(Z)-β-ocimene**	1032	1027	1235	1233	**3.37**	±0.35
**8**	**Linalool**	1095	1094	1543	1540	**5.75**	±1.64
**9**	2-ethyl-1,1-dimethyl-3 methylene-cyclohexene	-	1109	-	1561	0.57	±0.09
**10**	Bornyl acetate	1284	1274	1580	1575	0.94	± 0.38
**11**	β-elemene	1389	1386	1591	1588	1.97	±0.81
**12**	E-caryophyllene	1417	1404	1598	1592	0.87	±0.31
**13**	Germacrene D	1480	1472	1710	1703	0.87	±0.13
**14**	δ-cadinene	1522	1498	1756	1748	0.43	±0.18
**15**	(E)-γ-bisabolene	1577	1569	1745	1750	1.11	±0.31
**16**	Caryophyllene oxide	1582	1573	1987	1981	2.94	±1.06
**17**	Viridiflorol	1592	1595	2090	2082	1.13	±0.37
**18**	τ-muurolol	1640	1639	2186	2179	0.97	±0.27
**19**	α-cadinol	1652	1650	2227	2219	2.70	±0.67
**20**	**Cyclocolorenone**	1759	1748	-	2321	**45.34**	±2.48
**Class**			**Total (%)**			**99.47**	
Hydrocarbon monoterpenes			33.88				
Oxygenated monoterpenes			5.75				
			**39.63**				
Hydrocarbon sesquiterpenes			4.14				
Oxygenated sesquiterpenes			54.20				
			**58.34**				
Others			**1.51**				
Not identified			**0.53**				

Note: AI ^Teo^: Theoretical arithmetic retention index. AI ^Exp^: Experimental arithmetic retention index. S.D.: Standard deviation of triplicate analysis. Compounds were identified according to the essential oil database (Adams, 2017 [11]), NIST 2014 [21]. Total area presented as total ion counts (TIC). Names in bold correspond to major compounds and their relative percentage in the essential oil.

**Table 3:** Chemical composition of *Magnolia pugana* seeds essential oil (MpSEO) analyzed by GC/MS.

No.	Compound	Apolar DB-5 Column		DB-Wax Column		Total (%)	S.D.
		AI ^Teo^	AI ^Exp^	AI ^Teo^	AI ^Exp^		
**1**	α-pinene	932	949	1025	1022	1.07	±0.15
**2**	**β-pinene**	974	1003	1110	1107	**7.58**	±0.90
**3**	Isobutyl isovalerate	1014	1013	1294	1220	0.13	±0.05
**4**	β-elemene	1389	1395	1591	1510	1.97	±0.07
**5**	α-gurjunene	1409	1407	1529	1532	0.86	±0.04
**6**	E-caryophyllene	1417	1420	1598	1581	1.27	±0.04
**7**	α-bermagotene	1437	1442	1575	-	0.28	±0.03
**8**	α-humulene	1452	1459	1667	1654	0.26	±0.01
**9**	Germacrene D	1480	1484	1709	1703	0.48	±0.02
**10**	**α-selinene**	1495	1499	1725	-	**6.30**	±0.03
**11**	δ-cadinene	1522	1525	1756	1748	1.08	±0.02
**12**	(E)-γ-bisabolene	1500	1528	1745	1750	1.36	±0.09
**13**	cis,trans-nerolidol	1533	1530	2007	-	0.16	±0.06
**14**	**(I3)-caryophyleine**	1538	1539	-	-	**10.84**	±0.15
**15**	E-nerolidol	1561	1573	2036	-	0.58	±0.03
**16**	Longipinocarvone	1561	1575	-	-	2.87	±0.32
**17**	**Germacrene B**	1566	1582	1824	-	**5.52**	±0.09
**18**	Germacrene D- 4-ol	1574	1584	2057	-	1.17	±0.02
**19**	Caryophyllene oxide	1582	1587	1986	1981	1.80	±0.01
**20**	Ledol	1590	1610	2039	2027	0.73	±0.01
**21**	β-cedren-9-one	1630	1617	-	-	3.30	±0.03
**22**	Allo-aromadendrene epoxide	1639	1632	-	-	0.15	±0.00
**23**	(5β,7β,10β)-3,11-eudesmadien-2-one	1640	1639	-	-	0.16	±0.01
**24**	τ-muurolol	1640	1654	2186	2179	0.55	±0.10
**25**	Ent-spathulenol	1577	1659	2133	2125	0.27	±0.17
**26**	α-cadinol	1652	1666	2227	2219	1.07	±0.01
**27**	Helifolenol A	-	1684	-	-	2.22	±0.01
**28**	8-cedren-13-ol	1688	1698	-	-	0.44	±0.00
**29**	Eudesm-7(11)-en-4-ol	1700	1705	2282	2285	1.12	±0.01
**30**	14-hydroxy-α-humulene	1690	1714	-	-	0.22	±0.01
**31**	Isobicyclogermacrenal	1733	1740	-	-	0.16	±0.00
**32**	Eremophilone	1734	1744	-	-	1.78	±0.05
**33**	**Cyclocolorenone**	1759	1767	-	2321	**39.03**	±1.15
**34**	Dehidrosaussurea lactone	1838	1833	-	2401	0.40	±0.04
**Class**			**Total (%)**			**97.20**	
Hydrocarbon monoterpenes			10.62				
Oxygenated monoterpenes			0.86				
			**11.48**				
Hydrocarbon sesquiterpenes			32.17				
Oxygenated sesquiterpenes			53.42				
			**85.59**				
Others			**0.13**				
Not identified			**2.80**				

Note: AI ^Teo^: Theoretical arithmetic retention index. AI ^Exp^: Experimental arithmetic retention index. S.D.: Standard deviation of triplicate analysis. Compounds were identified according to the essential oil database (Adams, 2017 [11]), NIST 2014 [21]. Total area presented as total ion counts (TIC). Names in bold correspond to major compounds and their relative percentage in the essential oil.

**Table 4:** Chemical composition of *Magnolia pugana* flowers essential oil (MpFEO) analyzed by GC/MS.

No.	Compound	Apolar DB-5 Column		DB-Wax Column		Total (%)	S.D.
		AI ^Teo^	AI ^Exp^	AI ^Teo^	AI ^Exp^		
**1**	α-pinene	932	951	1025	1022	0.34	±0.10
**2**	β-pinene	974	1008	1110	1107	2.21	±0.58
**3**	(Z)-β-ocimene	1032	1064	1234	1230	0.68	±0.09
**4**	(E)-β-ocimene	1044	1075	1250	1233	0.77	±0.36
**5**	Cis-linalool oxide	1170	1185	1446	1428	0.19	±0.05
**6**	**β-elemene**	1389	1392	1591	1510	**3.85**	±0.60
**7**	α-gurjunene	1409	1404	1529	1532	0.61	±0.05
**8**	E-caryophyllene	1417	1418	1598	1592	2.62	±0.19
**9**	α-humulene	1452	1456	-	1654	0.50	±0.06
**10**	E-germacrene D	1480	1482	1708	1703	2.07	±0.96
**11**	β-selinene	1489	1488	1716	1709	0.41	±0.35
**12**	α-selinene	1498	1495	1725	1712	1.21	±0.12
**13**	Benzyl tiglate	1497	1500	-	-	0.29	±0.19
**14**	β-bisabolene	1505	1512	1727	1721	0.77	±0.30
**15**	δ-cadinene	1511	1521	1755	1748	0.91	±0.10
**16**	(E)-γ-bisabolene	1528	1531	1744	1750	0.83	±0.02
**17**	10-isopropenyl-3,7- cyclodecadien-1-one	1533	1534	-	-	1.39	±0.20
**18**	Elemol	1548	1559	2036	2013	0.27	±0.19
**19**	E-nerolidol	1561	1570	2078	2022	0.31	±0.02
**20**	**Caryophyllene oxide**	1582	1583	1986	1981	**2.65**	±0.00
**21**	Ledol	1602	1606	2039	2027	0.56	±0.08
**22**	γ-gurjunenepoxide-(2)	1626	1622	-	-	0.37	±0.28
**23**	Allo-aromadendrene epoxide	1639	1631	-	-	0.26	±0.10
**24**	Cis-guaia-3,9-dien-11-ol	1648	1644	-	-	0.10	±0.05
**25**	τ-muurolol	1640	1650	2186	2179	0.95	±0.10
**26**	Ent-spathulenol	1577	1655	2133	2125	0.69	±0.32
**27**	α-cadinol	1652	1662	2227	2219	1.53	±0.35
**28**	14-hydroxycaryophyllene	1668	1678	-	-	0.89	±0.67
**29**	Helifolenol A	1674	1684	-	-	1.35	±0.17
**30**	8-cedren-13-ol	1688	1694	-	-	0.25	±0.17
**31**	Eudesm-7(11)-en-4-ol	1700	1700	2300	2285	0.47	±0.13
**32**	14-hydroxy-α-humulene	1713	1710	-	-	0.35	±0.02
**33**	**2Z,6E-farnesol**	1722	1727	2324	2302	**8.47**	±0.69
**34**	Isobicyclogermacrenal	1733	1735	-	-	0.42	±0.20
**35**	**Cyclocolorenone**	1759	1764	-	2321	**41.95**	±1.72
**36**	Benzyl benzoate	1759	1774	2612	2584	1.12	±0.04
**37**	Epi-cyclocolorenone	1774	1781	-	-	0.12	±0.00
**38**	2α-acetoxyamorfa-4,7(11)-diene	1805	1818	-	-	0.38	±0.09
**39**	**Benzoic acid (5,5-dimethyl-4-oxo- 2-cyclohexenyl)-ester**	1874	1862	-	-	**8.41**	±0.21
**Class**			**Total (%)**			**91.51**	
Hydrocarbon monoterpenes			3.99				
Oxygenated monoterpenes			0.19				
			**4.18**				
Hydrocarbon sesquiterpenes			13.78				
Oxygenated sesquiterpenes			70.37				
			**84.15**				
Others			**3.19**				
Not identified			**8.49**				

Note: AI ^Teo^: Theoretical arithmetic retention index. AI ^Exp^: Experimental arithmetic retention index. S.D.: Standard deviation of triplicate analysis. Compounds were identified by MS, and according to the essential oil database (Adams, 2017) [11], NIST 2014 [21]. The total is presented as total ion counts (TIC). Names in bold correspond to major compounds and their relative percentage in the essential oil.

**Table 5:** *M. pugana* essential oils antioxidant activity on DPPH and ABTS radicals.

	DPPH ^1^		ABTS ^1^	
	IC_50_	S.D.	IC_50_	S.D.
MpLEO	37.40 ^c^	±4.60	14.80 ^c^	±4.20
MpSEO	21.50 ^b^	±4.50	17.40 ^c^	±1.20
MpFEO	27.90 ^b^	±2.80	9.04 ^b^	±0.56
BHT	1.66 ^a^	±0.15	0.24 ^a^	±0.02

Note: ^1^ DPPH and ABTS free radical scavenging activity expressed as IC_50_ value (mg/mL). Data presented as the mean ± S.D., *n* = 3. The means in each column followed by a letter show significant differences (*p* < 0.05).

**Table 6:** *M. pugana* essential oils antioxidant fractions isolated from a bioautographic assay with DPPH and identified by GC-MS.

Fraction	f1	f2	f3
*Rf*	0.97	0.79	0.56
MpLEO	β-elemene E-caryophyllene Germacrene D (E)-γ-bisabolene	Caryophyllene oxide τ-muurolol	-
MpSEO	β-elemene α-gurjunene E-caryophyllene Germacrene D (E)-γ-Bisabolene Ledol 8-cedren-13-ol	Longipinocarvone, Caryophyllene oxide τ-Muurolol Ent-spathulenol	α-selinene (I3)-Caryophyleine Germacrene B β-Cedren-9-one Eudesm-7(11)-en-4-ol 14-hydroxy-α-humulene
MpFEO	β-elemene α-gurjunene E-caryophyllene E-germacrene D β-bisabolene (E)-γ-bisabolene Ledol 8-cedren-13-ol 2α-acetoxyamorpha-4,7(11)-diene	Caryophyllene oxide τ-muurolol Ent-spathulenol	Eudesm-7(11)-en-4-ol 14-hydroxy-α-humulene

Note: Compounds analyzed and identified by GC-MS. *Rf* of fractions with antibacterial activity was calculated from the origin to 1 cm, and the solvent distance from the origin was 8 cm.

**Table 7:** *M. pugana* essential oils minimum inhibitory concentrations against pathogenic bacteria.

		MIC (mg/mL)			
Bacteria	Strain	MpLEO	MpSEO	MpFEO	Ampicillin ^1^
*E. coli*	ATCC^®^ 25922	22.73 ^b^	22.73 ^b^	22.73 ^b^	5.01 × 10^−3 a^
*E. coli*	ATCC^®^ 8739	22.73 ^b^	22.73 ^b^	22.73 ^b^	5.01 × 10^−3 a^
*K. pneumoniae*	ATCC^®^ 10031	5.68 ^c^	2.84 ^b^	5.68 ^c^	8.01 × 10^−2 a^
*S. typhimurium*	ATCC^®^ 14028	11.36 ^b^	11.36 ^b^	11.36 ^b^	2.51 × 10^−3 a^
*S. aureus*	ATCC^®^ 6538p	1.42 ^c^	0.71 ^b^	0.71 ^b^	6.30 × 10^−4 a^
*S. epidermidis*	ATCC^®^ 14990	0.71 ^c^	0.35 ^b^	0.71 ^c^	1.25 × 10^−3 a^
*B. subtilis*	ATCC^®^ 6633	11.36 ^b^	11.36 ^b^	11.36 ^b^	5.01 × 10^−3 a^

Note: ^a–c^ Different letters show differences between treatments (horizontal). ^1^ Positive control: Ampicillin.

**Table 8:** Inhibitory concentration of M. pugana essential oils against MCF-7 y HT-29 cell lines.

Cell line	MCF-7 (ATCC^®^ HTB-22)		HT-29 (ATCC^®^ HTB-38)	
Tested Agent	IC_50_	S.D.	IC_50_	S.D.
MpLEO	27.25 ^a^	±3.80	377.6 ^c^	±87.80
MpSEO	36.26 ^a^	±5.02	54.01 ^a^	±1.72
MpFEO	98.87 ^c^	±15.00	134.8 ^b^	±4.00
Cyclophosphamide ^1^	51.56 ^b^	±8.31	59.98 ^a^	±2.58

Note: Cytotoxic activity is expressed as IC_50_ value (µg/mL). The averages in each column followed by a letter show significant differences (*p* < 0.05). ^1^ Positive control. S.D.: Standard deviation.

## Data Availability

Data is contained within the article/supplementary material.

## Supplementary Materials

The following supporting information can be downloaded at: https://www.mdpi.com/article/10.3390/molecules30183778/s1, S1: Chromatographic profiles of the essential oils from leaves, seeds, and flowers of *M. pugana*, S2: Major compounds in the essential oils of *M. pugana* structure and mass spectra according to (Stein y Mirokhin, 2002), (NIST, 2018) [66,67].

## Abbreviations

The following abbreviations are used in this manuscript:

EOs	Essential oils
MpEOs	*Magnolia pugana* essential oils
MpLEO	*Magnolia pugana* leaf essential oil
MpFEO	*Magnolia pugana* flower essential oil
MpSEO	*Magnolia pugana* seed essential oil
TLC	Thin Layer chromatography
GC-FID	Gas chromatography-flame ionization detector
GC-MS	Gas chromatography coupled to mass spectrometry
MIC	Minimal inhibitory concentration
IC_50_	Inhibitory concentration 50%
TLC-DB	Thin Layer chromatography-Direct Bioautography
