# Supplementary material for: Chemical Composition and Bioactivity of Essential Oils from Magnolia pugana, an Endemic Mexican Magnoliaceae Species
Source: Molecules. 2025 Sep 17;30(18):3778. doi: 10.3390/molecules30183778 (PMC12473070; doi:10.3390/molecules30183778)
Supplement: Supplementary file 1 [file molecules-30-03778-s001.zip › molecules-3806165-supplementary.pdf]

**Supplementary Material**

S1: Chromatographic profiles of the essential oils from leaves, seeds, and flowers of *M. pugana*

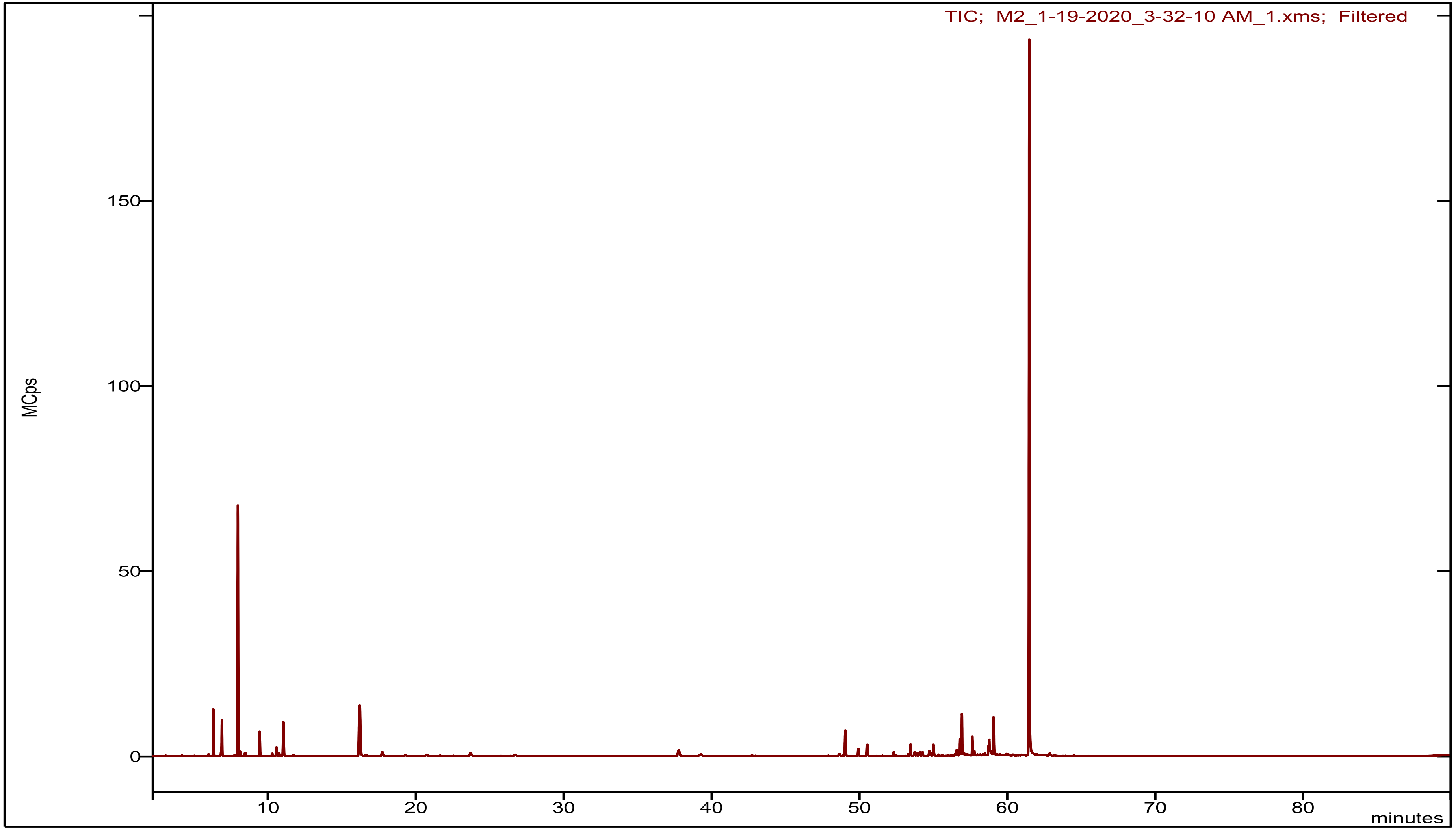


**Note:** Chromatogram of *M. pugana* leaves essential oil (MpLEO), injection volume 2 μL.

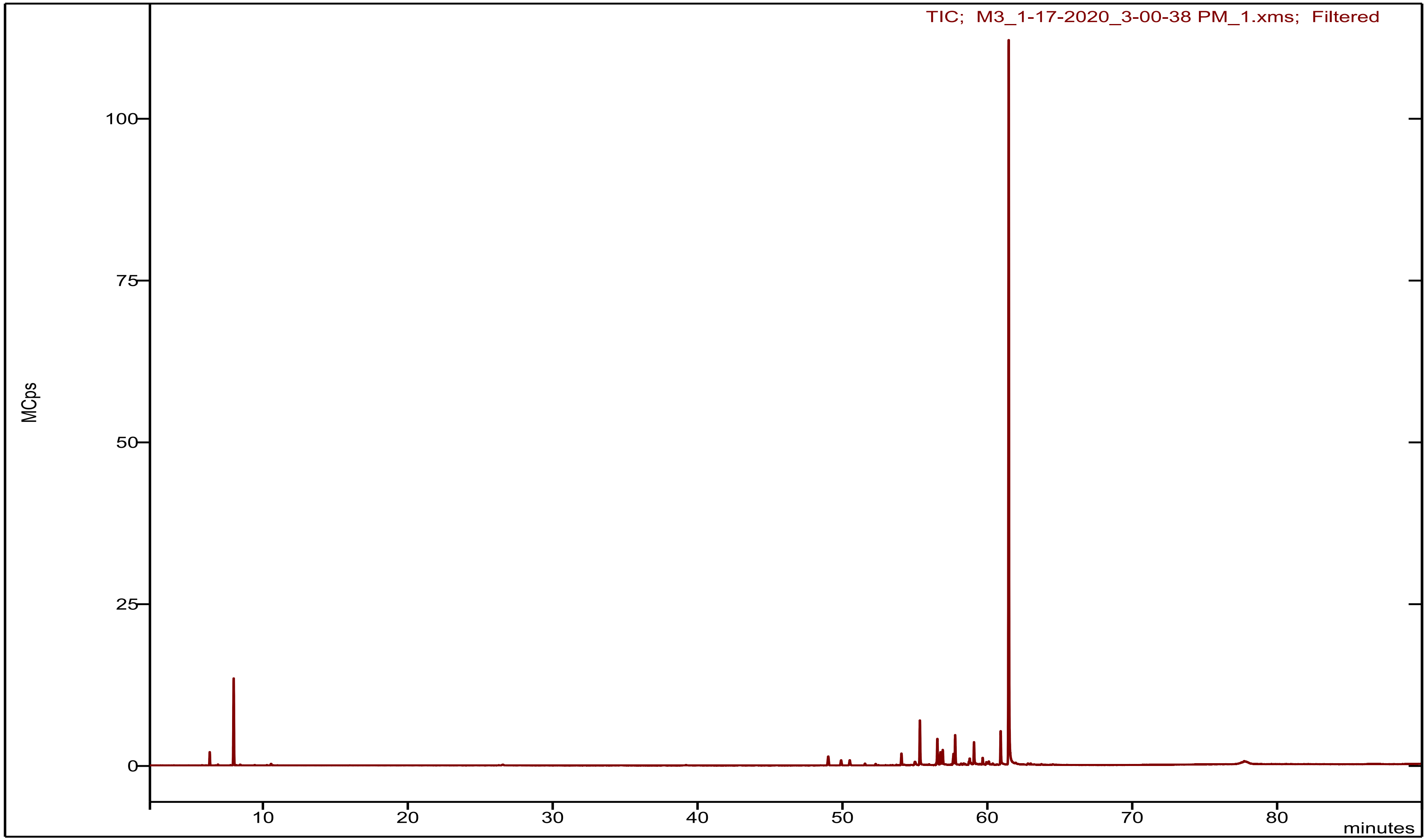


**Note:** Chromatogram of *M. pugana* seeds essential oil (MpSEO), injection volume 2 µL.

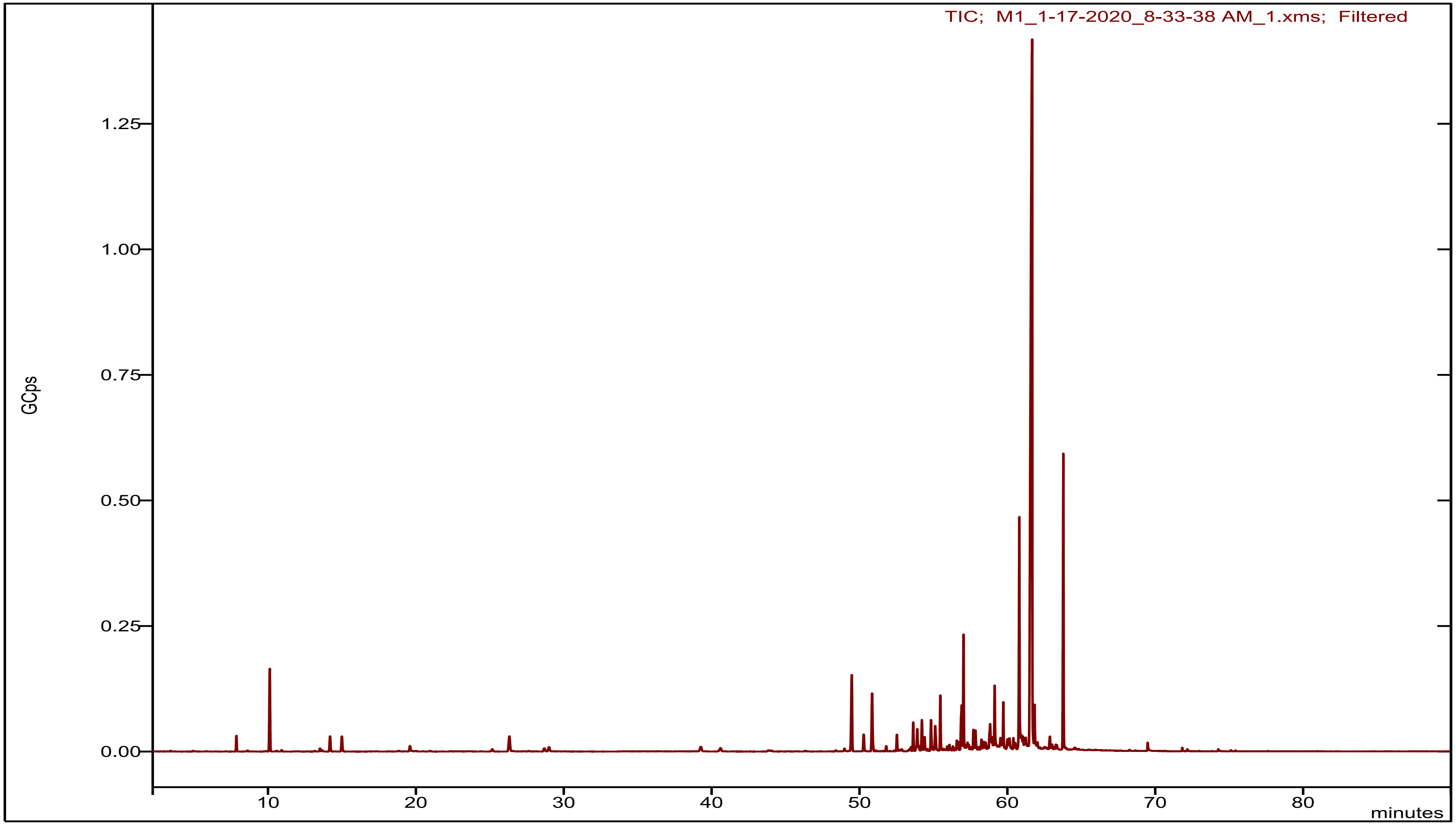


**Note:** Chromatogram of *M. pugana* flowers essential oil (MpFEO), injection volume 2 µL.

S2: Major compounds in the essential oils of *M. pugana* structure and mass spectra according to (Stein y Mirokhin, 2002), (NIST, 2018).

**A. MpLEO**

**Compound** | **Chemical structure** | **Mass spectrum**

**Cyclocolorenone**

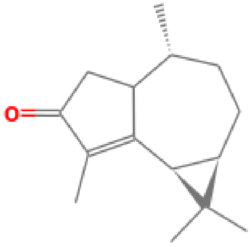


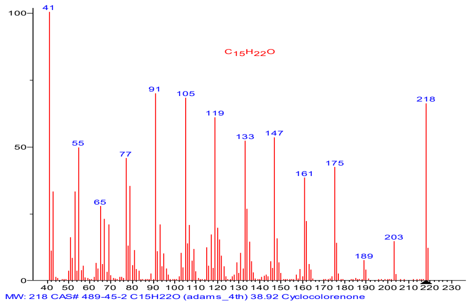


**β-Pinene**

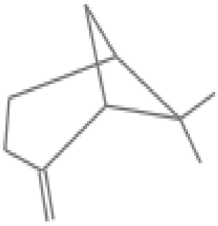

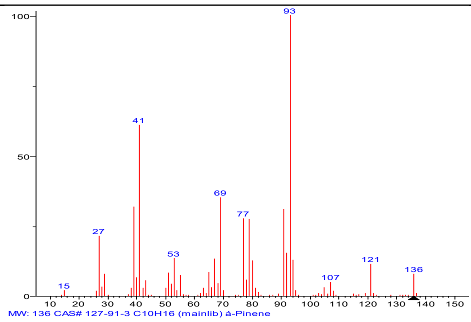


**Linalool**

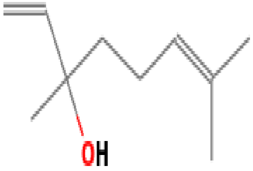


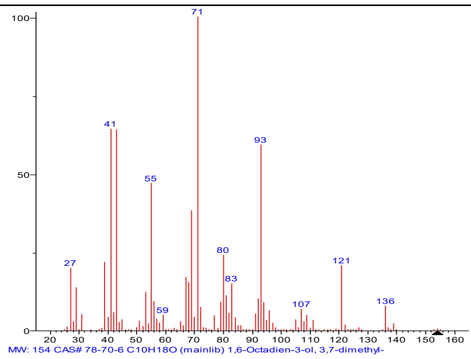


**α-Pinene**

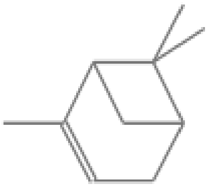

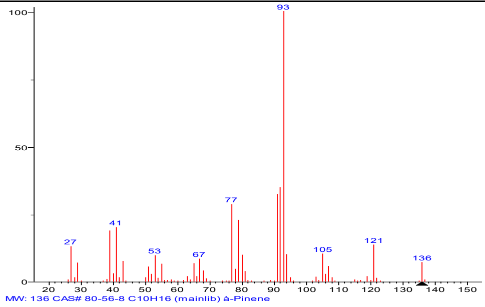


**(Z)-β-Ocimene**

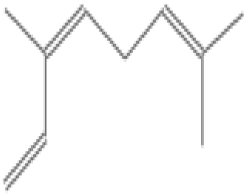

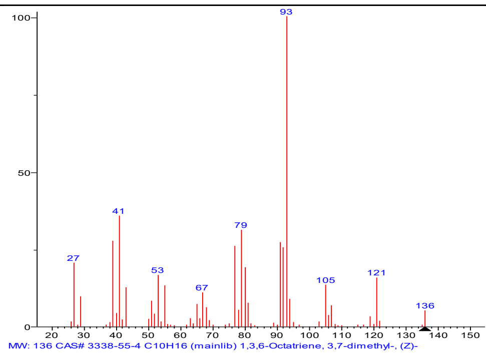


(Stein y Mirokhin, 2002), (NIST, 2018).

**B. MpSEO**

| Compound | Chemical structure | Mass spectrum |
| --- | --- | --- |
| **Cyclocolorenone** | 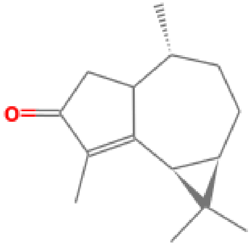 | 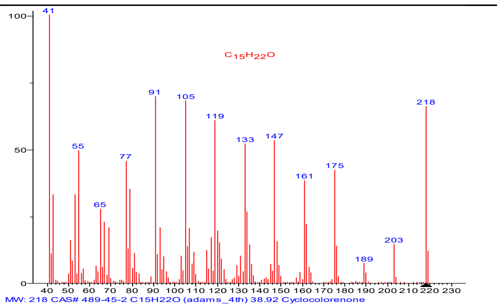  |
| **Caryophylleene-(I3)** | 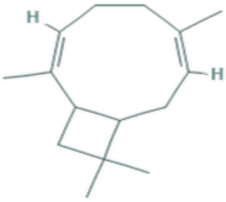 | 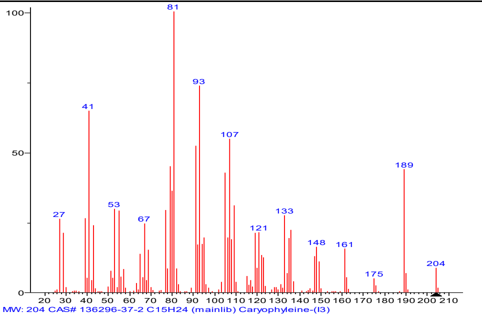  |
| **β-Pinene** | 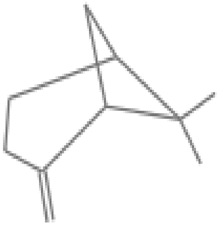 | 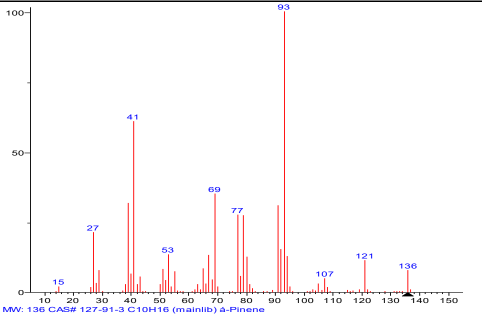  |
| **α-Selinene** | 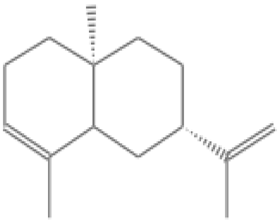 | 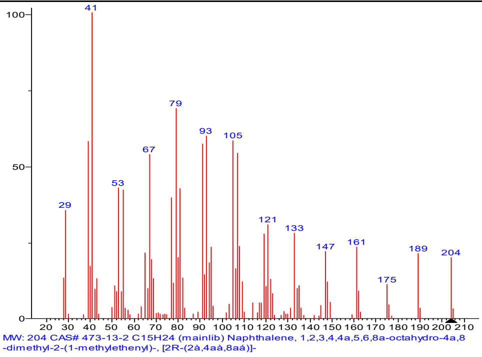  |
| **Germacrene B** | 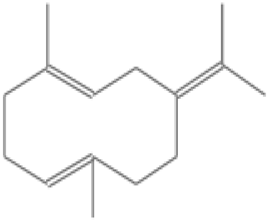 | 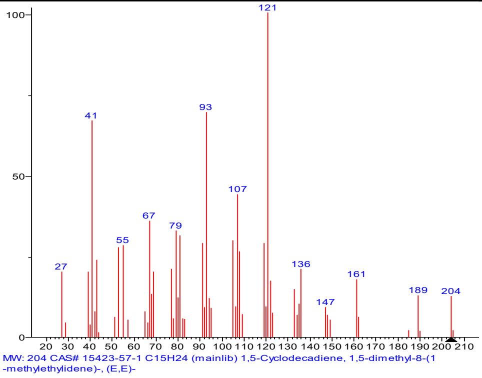  |

(Stein y Mirokhin, 2002), (NIST, 2018).

**C. MpFEO**

| Compound | Chemical structure | Mass spectrum |
|---|---|---|
| **Cyclocolorenone** | 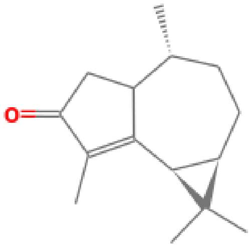 | 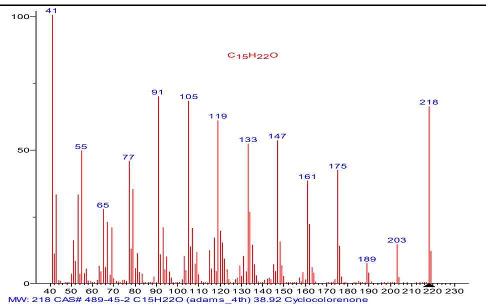 |
| **(Z,Z)-2,6-Farnesol** | 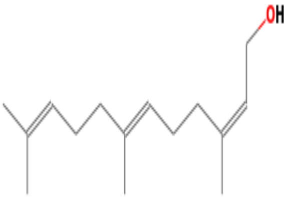 | 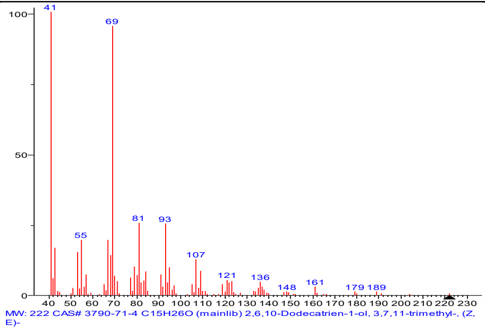 |
| **(5,5-Dimethyl-4-oxo-2-cyclohexenyl) benzoic acid ester** | 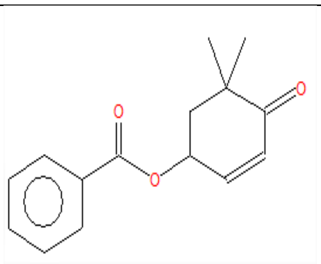 | 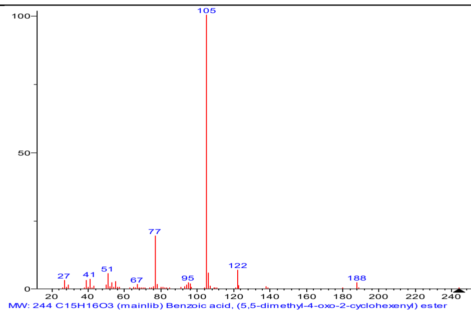 |
| **β-Elemene** | 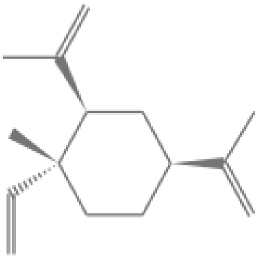 | 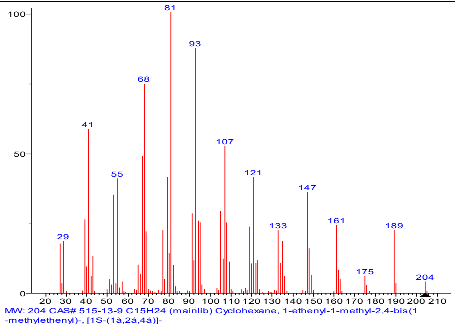 |
| **Caryophyllene oxide** | 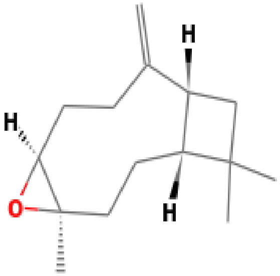 | 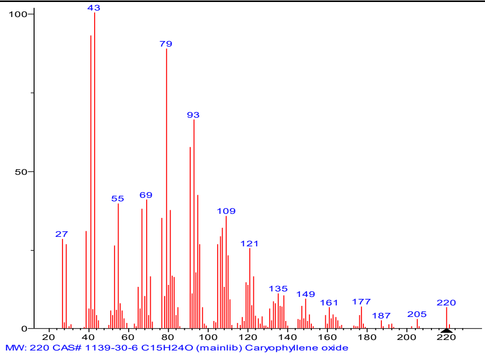 |

(Stein y Mirokhin, 2002), (NIST, 2018).
